# Chilaiditi Sign: When a “Free Air” Scare Turned Out to Be Chilaiditi’s Curious Cameo

**DOI:** 10.7759/cureus.89723

**Published:** 2025-08-10

**Authors:** Vimalraj Periyasami, Adithya Venkatasubramanian, Ameena Ismail, Serene Sidhique, Benedict Sebastiampillai

**Affiliations:** 1 Acute Medicine, Peterborough City Hospital, Peterborough, GBR; 2 Trauma and Orthopaedics, Pilgrim Hospital, Boston, GBR; 3 General Medicine, Karuna Medical College, Palakkad, IND

**Keywords:** air under the diaphram on chest x-ray, ct imaging, gastric perforation, radio anatomy, x-ray

## Abstract

Chilaiditi sign, a rare radiological finding of colonic interposition between the liver and diaphragm, can mimic pneumoperitoneum and lead to diagnostic confusion. We present the case of an elderly male who was initially treated for a transient ischemic attack and heart failure, discharged in stable condition, and later recalled due to a delayed radiology report suggesting free air under the diaphragm. An urgent CT scan excluded gastrointestinal perforation and confirmed the presence of the Chilaiditi sign. The scan also revealed an incidental mass in the mesentery that was later identified through further investigations as a neuroendocrine tumor, which turned out to be the underlying cause of the Chilaiditi configuration.

## Introduction

The Chilaiditi sign refers to the radiological appearance of the bowel interposed between the liver and right hemidiaphragm. The condition was first described by Demetrius Chilaiditi in 1910 and remains a rare entity, with an estimated prevalence of 0.025% to 0.28% of chest and abdominal plain films [1]. While often asymptomatic, it can be mistaken for free intraperitoneal air, a hallmark of gastrointestinal perforation, on plain radiographs. This misinterpretation can lead to unnecessary investigations or surgical interventions. We report a case where the Chilaiditi sign was identified on a delayed chest X-ray report, triggering urgent reassessment for suspected pneumoperitoneum. Further imaging revealed that the small bowel was interposed between the liver and diaphragm, a finding that accounts for only 3-5% of Chilaiditi configurations [1]. Additionally, this scan incidentally detected a lesion, which was ultimately identified as the underlying cause of the Chilaiditi configuration.

## Case presentation

**Figure 1:**
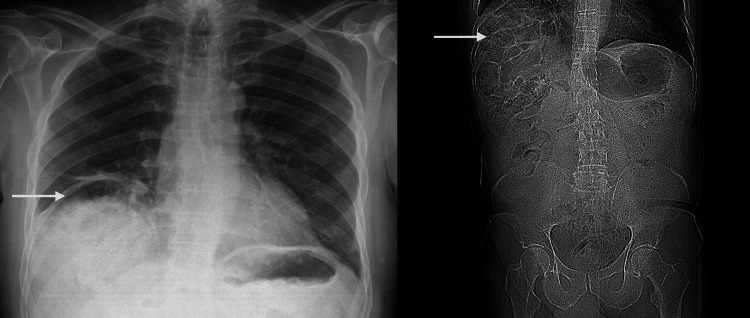
Chest X-ray and the scout image of the abdomen and pelvis CT, A gas-filled loop of colon is interposed between the liver and the right hemidiaphragm, mimicking free intraperitoneal air.

A 75-year-old male presented to the Medical Assessment Unit with symptoms consistent with a transient ischemic attack. During admission, he also had signs suggestive of heart failure, including bibasal crackles and peripheral edema, prompting a chest X-ray (Figure 1).

The X-ray was initially unreported during his inpatient stay. Subsequently, the delayed X-ray report was issued as a Red Star alert, indicating an urgent finding. It described elevation of the right hemidiaphragm with gas underneath, raising suspicion for pneumoperitoneum. The consulting physician was notified, and the patient was urgently recalled for a contrast-enhanced CT of the abdomen and pelvis. Clinical history revealed no abdominal pain, no change in bowel habits, no distention, no nausea and vomiting, and no shoulder tip pain. On examination, there was no tachycardia or hypotension, normal bowel sounds, and no rebound tenderness or guarding. Blood tests, including the liver function test, were unremarkable. Lactate and urea were within normal limits. Despite this, an urgent CT was planned, given that the perforation may be silent or present atypically in elderly patients.

**Figure 2:**
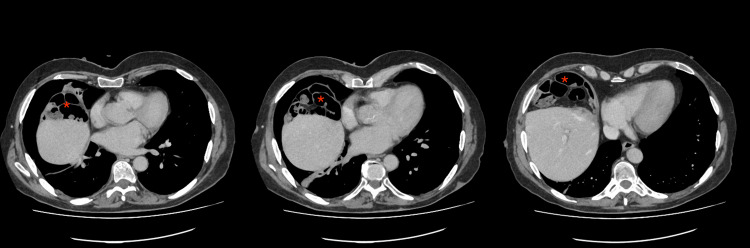
Axial CT showing the Chilaiditi sign: interposition of the colon between liver and diaphragm. The asterisk (*) marks the bowel segment situated above the liver.

The CT scan (Figure 2) revealed no free gas but identified small bowel loops interposed between the liver and diaphragm, consistent with the Chilaiditi sign. CT of the abdomen also demonstrated a 35 mm partly calcified mesenteric mass of uncertain etiology. Additionally, linear low attenuation noted high in the right liver lobe was interpreted to represent dilated bile ducts. Biochemical correlation was recommended, along with consideration of MRI of the liver. Considering the benign nature of the Chilaiditi sign, the patient was discharged home with further follow-up scans for the incidental findings.

**Figure 3:**
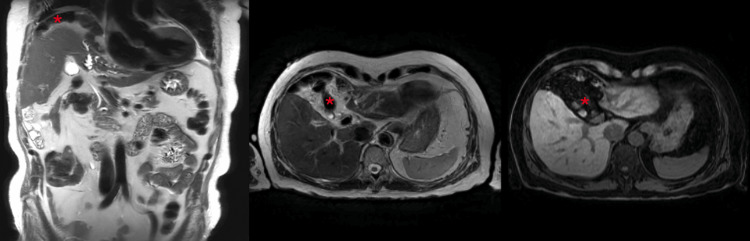
MRI performed as a follow-up of the CT scan confirming the Chilaiditi sign. The asterisk (*) marks the bowel segment situated above the liver.

These findings were discussed at the Colorectal Multidisciplinary Team meeting (MDT), which recommended MRI of the liver and magnetic resonance cholangiopancreatography (MRCP) for further evaluation. MRI of the liver (Figure 3) also confirmed that the bowel was interposed between the right hemidiaphragm and the anterior surface of the liver in Chilaiditi configuration with resultant scalloping and distortion of the liver contour. MRCP and MRI of the liver also showed progressive atrophy of the left lobe of the liver since 2016, with new moderate left lobar ductal dilatation secondary to a left hepatic duct stricture with no evidence of a hepatic mass. Liver function test also showed chronically elevated alkaline phosphatase with other parameters, including aspartate aminotransferase, bilirubin, and total proteins being normal. Alpha-fetoprotein (AFP) levels were also normal.

Referral to the Hepatobiliary MDT was recommended, and the MDT suggested positron emission tomography/computed tomography (PET/CT) and cancer antigen 19-9 (CA 19-9). PET/CT impression was that the liver was optimally assessed by MRI, and there was no feature of fluorodeoxyglucose (FDG)-avid nodal or distant metastasis. Additionally, left of midline mesenteric part, a calcified mass adjacent to the small bowel was confirmed to be FDG-avid. In the setting of neurofibromatosis, this may represent a neuroendocrine tumor (NET). CA 19-9 was elevated at 41 U/mL, and a normal AFP was noted. Post MDT discussion, a working diagnosis of NET was made, but on clinic review, the patient was deemed not fit for intervention. Hence, a gallium PET scan or octreotide scan was planned before considering lanreotide.

The Chilaiditi sign was first identified on CT. While MRI and PET were performed to evaluate incidental liver findings, they incidentally confirmed the interposition as well. These follow-up scans were not aimed at assessing the Chilaiditi Sign but are mentioned here to highlight the consistency of the finding. This also highlights the important point that the atrophied liver was a result of the bile duct stricture caused by the NET, which, in turn, led to reduced liver volume and the development of the Chilaiditi configuration.

## Discussion

The Chilaiditi sign is an uncommon but clinically important radiological finding characterized by the interposition of a segment of bowel, most commonly, the ascending colon, hepatic flexure, and transverse colon, between the diaphragm and liver. On the other hand, as in our case, only 3-5% of cases with small intestine involvement have been described. This anatomical variant can mimic pneumoperitoneum, leading to diagnostic confusion and sometimes unnecessary surgical intervention. When this radiological finding is accompanied by clinical symptoms, it is termed Chilaiditi syndrome [2]. It is more commonly seen in older adults, particularly males, and is often asymptomatic, being discovered incidentally during radiographic evaluation for unrelated conditions. In our case, the finding was initially incidental, discovered only after retrospective review of a chest X-ray that had been delayed in reporting.

This scenario highlights one of the most significant clinical concerns with the Chilaiditi sign: its radiological resemblance to pneumoperitoneum. True pneumoperitoneum, typically resulting from gastrointestinal perforation, presents with free intraperitoneal air visible under the diaphragm on upright imaging. This can lead to emergency laparotomy if not correctly identified. Chilaiditi sign, however, represents intraluminal bowel gas trapped between the liver and diaphragm, not free peritoneal air [2,3]. Differentiating between the two is critical.

Several radiological features can help differentiate the Chilaiditi sign from true pneumoperitoneum: [4] (1) the haustral pattern of the interposed colon may be visible on plain radiographs; (2) the gas lucency does not change with patient positioning; and (3) the absence of peritoneal signs clinically should prompt reevaluation before invasive action.

In our case, an urgent CT scan of the abdomen and pelvis with contrast was instrumental in confirming the diagnosis. It revealed loops of small bowel interposed between the liver and right hemidiaphragm, with no evidence of free air or perforation, definitively ruling out pneumoperitoneum. CT is considered the gold standard in distinguishing the Chilaiditi sign from life-threatening causes of pneumoperitoneum, as it allows clear visualization of anatomical relationships and air patterns [2].

The exact cause of the Chilaiditi sign is not fully understood, but multiple predisposing factors have been identified including laxity or absence of suspensory ligaments (e.g., falciform ligament), redundant or abnormally long colon, chronic constipation or abdominal distension, diaphragmatic elevation or phrenic nerve paralysis, reduced liver volume (e.g., in cirrhosis), chronic obstructive pulmonary disease and other causes of hyperinflation, ascites, or obesity [3]. In this case, there was an established long-standing progressive atrophy of the left lobe of the liver, as evidenced by the MRI of the liver. Our patient had a background of neurofibromatosis, hydrocephalus, and previous neurosurgical interventions, but no hepatic or pulmonary conditions diagnosed previously, nor signs of intestinal obstruction. The incidental nature of the finding is consistent with the asymptomatic Chilaiditi sign, though the event did provoke a cascade of concern due to its initial misinterpretation. The differential diagnosis for gas under the right hemidiaphragm in the plain film includes pneumoperitoneum (due to hollow viscus perforation), subphrenic abscess, diaphragmatic hernia, Morgagni hernia, volvulus, gas within intra-abdominal cysts, or abscesses [3]. In this case, the underlying cause was elucidated through follow-up MRI and PET/CT imaging. An FDG-avid mesenteric mass was identified and, when correlated with the patient’s known diagnosis of neurofibromatosis and supporting biochemical investigations, was diagnosed as a NET. The NET led to biliary stricture, which subsequently caused lobar hepatic atrophy. This reduction in liver volume allowed the small bowel to interpose between the liver and diaphragm, resulting in the Chilaiditi sign.

Misinterpreting the Chilaiditi sign can result in invasive diagnostics, unnecessary surgery, and prolonged hospital stays, not to mention patient anxiety. In the emergency setting, clinicians must rely not only on imaging but also on thorough clinical assessment, including abdominal examination, vital signs, and inflammatory markers. In this case, the absence of peritoneal signs, systemic toxicity, or abdominal pain should have lowered the suspicion of perforation. However, given the patient’s age and the possibility of atypical presentations of perforation in older adults, further evaluation with a CT scan was deemed appropriate. Asymptomatic Chilaiditi sign, as seen here, requires no specific treatment. However, symptomatic Chilaiditi syndrome may present with abdominal pain, nausea, vomiting, or even bowel obstruction. Initial management includes conservative measures such as bowel rest, fluid and electrolyte correction, and nasogastric decompression [5]. In rare, refractory cases, surgical interventions such as colopexy, hepatopexy, or bowel resection may be required [6].

## Conclusions

This case highlights a rare presentation of the Chilaiditi sign caused by small bowel interposition, which accounted for only a minority of such cases. Further imaging revealed an underlying FDG-avid mesenteric NET, diagnosed in the context of neurofibromatosis and supportive biochemical findings. The NET led to biliary stricture and subsequent lobar hepatic atrophy, ultimately facilitating the anatomical configuration necessary for the Chilaiditi sign to occur. This case underscores the importance of thorough investigation of incidental radiological findings, which may reveal clinically significant and otherwise unsuspected pathology. This case also underscores the critical importance of timely reporting and communication of imaging results, awareness of anatomical variants such as the Chilaiditi sign among emergency and general physicians, and the appropriate use of cross-sectional imaging before escalating care.
